# Satisfaction with online teaching of medical statistics during the COVID‐19 pandemic: A survey by the Education Committee of the Italian Society of Medical Statistics and Clinical Epidemiology

**DOI:** 10.1111/test.12286

**Published:** 2021-07-22

**Authors:** Matteo Rota, Giulia Peveri, Margherita Fanelli, Lucio Torelli, Marco BL Rocchi, Claudia Specchia

**Affiliations:** ^1^ Department of Molecular and Translational Medicine University of Brescia Brescia Italy; ^2^ Department of Clinical Sciences and Community Health University of Milan Milan Italy; ^3^ Department of Interdisciplinary Medicine “Aldo Moro” University of Bari Bari Italy; ^4^ Department of Medicine, Surgery and Health Sciences University of Trieste Trieste Italy; ^5^ Department of Biomolecular Sciences University of Urbino “Carlo Bo” Urbino Italy

**Keywords:** teaching, distance education, medical statistics, surveys and questionnaire, teaching statistics

## Abstract

On May 2020, after 2 months of online teaching with no face‐to‐face lectures, the Education Committee of the Italian scientific Society of Medical Statistics and Clinical Epidemiology conceived an online survey to assess satisfaction of Italian academics of medical statistics with online teaching and remote exams. This survey highlighted teachers' perceptions as well as opportunities and limitations of online teaching of medical statistics, biostatistics, and epidemiology. Although 61% of Italian academics of medical statistics declared to be favorable to provide online teaching of medical statistics, biostatistics, and epidemiology in the future, we recognize that distance education cannot substitute the unique value of teaching and knowledge exchange that could only be transmitted through a personal interaction between students and teachers. These indications may be useful to improve the quality of the teaching process in the future.

## 1 INTRODUCTION

On March 9, 2020, the Italian government imposed the first national lockdown in response to the pandemic of coronavirus disease 2019 (COVID‐19) in the country. This caused the closure of the universities, leading to the suspension of face‐to‐face classes and forcing distance learning to be the core method of teaching. Distance learning has its pros and cons, and its success depends on many factors, including accessibility, that is, availability of web access and a broadband connection, use of appropriate modalities, course contents, and assessment criteria for learning verification [1].

From the teacher's perspective, distance learning can be approached either by synchronous or asynchronous lessons or in a mixed‐mode. Clear indications about the modalities to deliver distance learning were often missing and varied a lot between academic institutions. Moreover, the choice of one modality over the other did not have the same weight within various degree courses and disciplines. None of the Italian public universities traditionally used distance learning and was prepared to approach it.

The Education Committee of the Italian scientific Society of Medical Statistics and Clinical Epidemiology (SISMEC) [14] has the mission to promote initiatives aimed at harmonizing the objectives and the teaching of medical statistics in tertiary education degree courses. With the suspension of face‐to‐face teaching activities, the Committee wondered at a national level about the comparison of methods for delivering online courses of medical statistics, biostatistics, and epidemiology, as well as possible tools for evaluating the students' acquired knowledge. In fact, a crucial role is played by modalities for carrying out online exams, including the use of proctoring software for students' remote control.

After 2 months of distance learning with no face‐to‐face lectures, in May 2020, the Committee conceived an online survey to be administered to the Italian academics with a twofold aim: on the one hand, to highlight the limitations of online teaching, and on the other hand, to highlight some opportunities that the situation had generated, including the degree of teachers' satisfaction.

## 2 THE PANORAMA OF MEDICAL STATISTICS TEACHING IN ITALIAN UNIVERSITIES

The teaching of medical statistics to undergraduate medical students has been the matter of discussion for long time, and it have leant toward application of the evidence‐based medicine (EBM) paradigm [12]. In Italy, medical statistics courses encompass the application of classical statistical knowledge to medical data as well as those in the wider field of health sciences. Topics comprise classical statistical concepts and methods such as descriptive statistics, data visualization, statistical inference, diagnostic accuracy, measures of association, and regression analysis with emphasis on study design. At the end of the course, students are expected to be able to observe and measure physical phenomena and develop statistical models to describe them.

The theoretical coverage of quantitative methods is often integrated by the development of practical technical skills in order to allow the analysis of real medical data using an appropriate statistical software.

Moreover, in accordance with the EBM paradigm, courses of medical statistics aim to provide students the knowledge for the critical appraisal of the published evidences in relation to their work context with special reference to clinical trials, epidemiology, and clinical or laboratory research.

## 3 METHODS

### 3.1 Survey development

This online survey, conceived by the Education Committee of the Italian scientific SISMEC [14], aimed to collect data on the provision and effectiveness of online teaching and exams of medical statistics, biostatistics, and epidemiology during the suspension of face‐to‐face didactic activities ordered by the decree of the Presidency of the Council of Ministers of February 23, 2020, to contain the effects of the COVID‐19 pandemic. Data have been collected anonymously, for the sole purpose of the research, in compliance with the EU General Data Protection Regulation no. 679/2016 and with the Italian Legislative Decree no. 196/2003 (amended by Legislative Decree no. 101 of 10.08.2018).

The survey was built using Microsoft Forms and was structured into four sections containing a total of 45 questions. The first part collected information about the academic role, age, and gender of the respondent. The second one included 21 questions on online teaching, ranging from the number of courses provided online, and the number of students expected to attend them, to the perceived teacher's satisfaction with online teaching. Similarly, the third part of the survey was focused on online exams and included 17 questions ranging from the modalities of delivery to the technical problems encountered during online exam sessions, to the perceived teacher's satisfaction. The final section of the survey included four questions on future perspectives of online teaching of medical statistics, biostatistics, and epidemiology. The full list of questions is reported in the Table S1.

### 3.2 Survey administration

On April 30, 2020, we accessed the online freely available official database of the Italian Ministry of Education, University and Research [9] in order to identify the list of Italian academics of medical statistics. The institutional e‐mail addresses of the identified academics were then retrieved either from the SISMEC members list and profiles or directly from their university's website.

On May 18, 2020, an e‐mail providing with information about the aims of the survey, and the URL link to access it, was sent out to the 122 Italian academics of medical statistics. No incentives were provided to complete the online questionnaire, which took approximately 10 minutes. A follow‐up e‐mail was sent out after 1 week, while a final reminder was sent 3 days before the closing date planned on May 31, 2020. The survey was also publicized on the SISMEC website [14] and through the society's newsletter in order to reach the adjunct professors who were not listed in the national database [9]. All responses were anonymous and confidential.

### 3.3 Statistical analysis

Categorical variables were described in terms of frequencies, while continuous variables were described in terms of median and range. Bar plots were used to graphically represent results from selected questions.

Satisfaction was defined with a score greater than or equal to 7/10 at the survey questions “From 1 to 10, how much did you enjoy delivering online teaching?” and “From 1 to 10, how much did you enjoy delivering online exams?,” respectively. Relative association measures were computed as ratio of satisfaction proportions within strata of factors influencing teachers' satisfaction with online teaching and online exams. Uncertainty among estimates was assessed through the 95% confidence intervals (CIs).

Analyses were carried out through SAS version 9.4 (SAS Institute Inc., Cary, North Carolina), and graphs were drawn with R version 4.0.2 (R Development Core Team).

## 4 RESULTS

A total of 94 questionnaires were completed by Italian academics of medical statistics, including five adjunct professors (Table A1). Among all the responders, 23 (24%) were full professors, 39 (41%) associate professors, 27 (29%) assistant professors, and 5 (5%) adjunct professors. Gender resulted fairly balanced with a slight predominance of females (54%).

The main items of the survey are summarized in Table S1. By the time the survey was sent out and completed, 88 academics (94%) had switched to online teaching, while only 37 (39%) declared a previous experience with online teaching.

The online teaching experience during the first months of the COVID‐19 pandemic was different with regards to the number of teaching hours, the number of courses, and the number of students. As for the latter point, the median of student numbers for the most attended course was 100. Educational provision varied across our sample: 21 (24%) chose to provide their lessons only asynchronously, 31 (35%) only synchronously, 18 (20%) synchronously but also provided asynchronous material, and 18 (20%) alternating the two modalities. Although technical difficulties during online teaching were experienced by 19 (22%) academics, a satisfaction score of 7 or more out of 10 was reached by 66 of them (75%).

**FIGURE 1:**
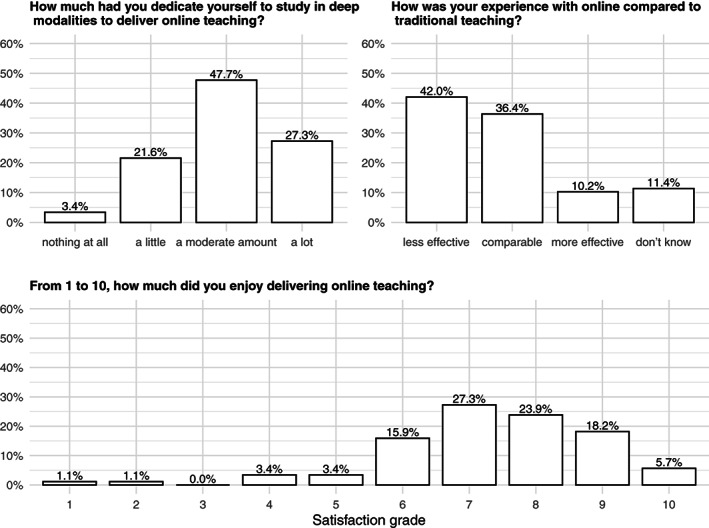
The experience with online teaching in a sample of Italian academics of medical statistics

Overall, the effort put into online teaching was high: three out of four academics declared they had dedicated themselves to study in deep online teaching modalities from a moderate amount to a lot. Nevertheless, 42% still found online teaching less effective than traditional modalities (Figure 1).

Among academics who had already delivered online exams at the time of the survey (73%), 20 (22%) choose an oral, 26 (29%) a written, and 37 (41%) a mixed (oral and written) modality. In the presence of large classes, it was necessary to organize multiple online rounds for the same exam. A satisfaction score of 7 or more out of 10 for online exams was declared by 40 academics (62%).

**FIGURE 2:**
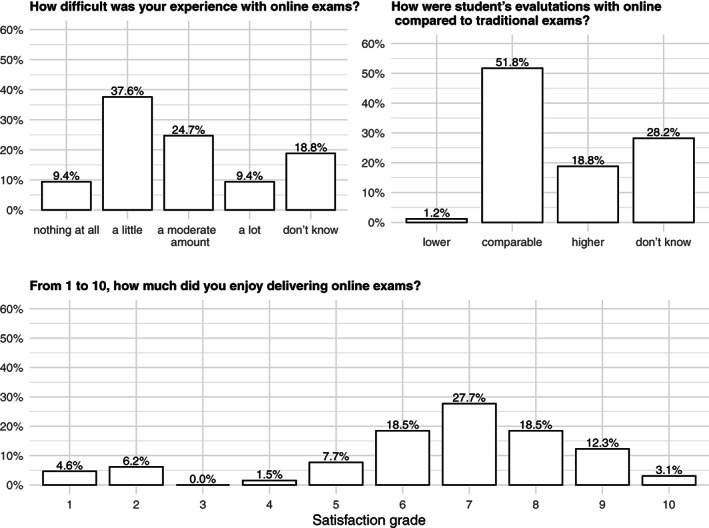
The experience with online exams in a sample of Italian academics of medical statistics

The experience with online exams was judged more difficult compared to the experience with online teaching, and relatively more negative evaluations were expressed (10% scores of 1 or 2 out of 10) (Figure 2). Students' evaluations with online compared to traditional exams were comparable for more than half of the sample (52%).

Association between teaching variables and satisfaction with online teaching is reported in Table A2.

Although there was no statistical significance, 67% of academics aged 40 or lower were satisfied with online teaching as compared to 87% of those aged 61 years or older. A slightly higher satisfaction was declared by males (78%) than females (72%). With reference to the academic role, 65% of full professors were satisfied as compared to 78% of associate professors and 76% of assistant professors.

Satisfaction was higher when the number of teaching hours and the amount of teaching courses increased.

Conversely, it was negatively influenced by an excessive number of students and technical difficulties.

The adoption of multiple teaching modalities has been judged as the most satisfactory solution.

Association between teaching variables and satisfaction with online exams is reported in Table A3.

Having experienced technical difficulties was strongly associated with a lower satisfaction. No other factor, among those considered, was found to be statistically significant, but indicators of more complex exam administration modalities, such as the need to create multiple rounds for the same exam and provision of both oral and written online exams, were inversely associated with satisfaction.

**FIGURE 3:**
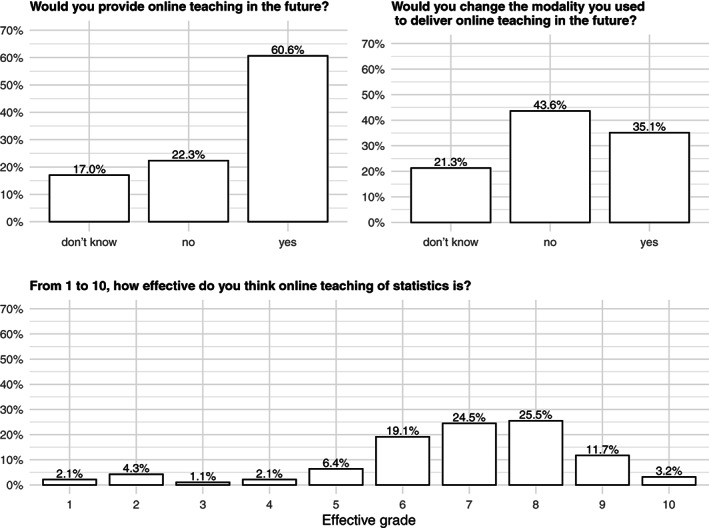
Perspectives of online teaching of medical statistics, biostatistics, and epidemiology in a sample of Italian academics of medical statistics

For what concerns future perspectives, 61% of the academics declared to be keen to provide again online teaching in the future and 35% would be open to explore different modalities for online teaching. Overall, 65% of them gave to their perceived effectiveness of online teaching of medical statistics, biostatistics and epidemiology a score of at least 7 out of 10 (Figure 3).

## 5 DISCUSSION

The COVID‐19 pandemic has changed our lives and determined a deep change in all educational activities. Although a year has already passed since the outbreak, at the time of writing (March 2020) higher tertiary education is still provided online in Italy and in other countries in the world. For this reason, a continuous assessment of teachers' opinions, students' needs, and critical points related to distance educational activities is mandatory to improve the quality of the teaching process [3].

The Education Committee of the Italian scientific SISMEC [14] has long been promoting initiatives aimed at harmonizing the objectives and the contents of medical statistics, biostatistics, and epidemiology courses in higher tertiary education, including Bachelor and Master of Science degree courses, postgraduate first and second‐level masters, specialization schools in the medical area and PhD courses. On May 2020, the Committee carried out a survey to evaluate the perception of Italian academics of medical statistics on online teaching and remote exams during the COVID‐19 pandemic. The response rate was 73%, showing a widespread interest toward issues related to online teaching of medical statistics and administration of remote exams.

Although only 39% of Italian academics declared a previous experience with online teaching before the suspension of in‐presence didactic activities due to the COVID‐19 pandemic, our survey showed a median satisfaction score with online teaching of 7 out of 10. Indeed, the survey showed that there was a balance between teachers who judged online teaching comparable or even more effective than in‐presence teaching as compared to those who did not. It has also to be pointed out that 75% of Italian academics of medical statistics had dedicated themselves to study in deep the modalities to provide online teaching, as they were forced by the COVID‐19 outbreak to move all teaching courses online without the possibility to properly design an online course, one of the most powerful factors impacting successful online learning outcomes [16]. Despite such a positive framework, some drawbacks were highlighted, including the lack of adequate personal interaction between students and teachers even when lessons were carried out through synchronous modalities [2]. The lack of a personal relationship also prevents teachers to get a feedback of students' learning. Several recent pieces of research [7, 10, 15] showed that online teaching increased gaps in students' success, resulting in a lower performance for those students with weak academic backgrounds who suffered most from the loss of the teacher–student interaction. In contrasts to these findings, our survey highlighted that student's performance was comparable (52%) or even higher (19%) in online as compared to in‐presence exams, in line with the findings by Ni [13] and with a review by Merisotis and Phipps [11]. The effectiveness of teaching statistics through a distance learning course as compared to a face‐to‐face course was a matter of discussion for a long time. A survey by Harrington [8] published more than 20 years ago suggested that students can learn statistics successfully through distance courses, but some students might need additional assistance or do better in a traditional format.

One of the success keys of online teaching lies in the easy way to quickly share educational materials to students through the University's learning management system (LMS). Previous published research showed that electronic delivery of course contents was associated with improved student outcomes [4]. Conversely, the most frequent problem was related to network connectivity. Several teachers experienced slow web connections or network problems, which led sometimes to a temporary suspension of synchronous lessons. For teachers who chose to deliver didactic activities through asynchronous modalities, difficulties arose during the upload of educational material, including lessons registrations, to the University's LMS, either due to a system overload or due to a slow internet connection.

Experience with remote exams was less satisfactory as compared to online teaching. Although the median satisfaction score with remote exams was 7 out of 10, more than 25% of Italian academics of medical statistics declared to be not fully satisfied. The most preferred modality to deliver remote exams was a combination of oral and online tests, the latter being administered either through the University's LMS or through other software. One in five teachers delivered oral exams only. However, the drawback of oral exams for large classes was the need to organize multiple online rounds for the same exam session. This translated into a full‐time engagement lasting several days for those academics involved in more than one course. Despite such evident limitation, satisfaction with remote exams did not appear to be related to the need of organizing multiple online rounds for the same exam. More interestingly, our survey showed that satisfaction with remote exams was significantly lower in those who experienced technical difficulties or problems. These difficulties included network connectivity issues, as well as the lack of teachers' knowledge on software to deliver remote exams. To this aim, the university's IT service has a pivotal role in the continuous dissemination of advancements and updates of didactic software and services available to teachers. Nowadays, university's LMSs allow to deliver written exams structured as online quiz using e‐proctoring services [6] to ensure remote control of students while taking examinations. Indeed, several academics were not aware of these advance functionalities at the time of the survey conduction.

Our survey had some limitations. First, it was administered to the Italian academics of medical statistics, a small community including only 122 among full, associate, and assistant professors at the time of the survey conduction. Although the sample was relatively small, the response rate (73%) was satisfactorily and similar across categories defined by the academic role. Second, we only got five replies from adjunct (contract) professors despite the survey has been publicized on the SISMEC website [14] and through the society's newsletter. In fact, Italian universities yearly enrolled several external teachers of medical statistics to cover all the didactic needs. Third, the survey was based on self‐reported data, and it was conducted within the COVID‐19 pandemic context. Fourth, although several academics highlighted an absence of interaction and human factor with students, the survey lacked quantification of the grade of interaction and also the use of specific audience interactions e‐platforms or University's LMSs to get students' involvement. These limitations may hamper the generalization of the findings.

Online teaching could have been initially considered only as a rescue modality to deliver educational activities during the COVID‐19 pandemic. As written by Gallo et al [5] in a recent report of the Education Committee of the Italian SISMEC, a change of education routines might be perceived as some loss of teacher identity [5]. However, the 61% of Italian academics declared to be favorable to provide online teaching of medical statistics, biostatistics, and epidemiology in the future, with a median perceived effectiveness of at least 7 out of 10. However, this positive view toward online teaching should be interpreted with caution, as it seems clear that technology cannot substitute the human factor [3, 4]. Today, 1 year and half after the start of the experience with online teaching in Italian universities, we could state that outcomes will improve over time as faculties and institutions progressively gain more experience. At the same time, we recognize that distance education cannot substitute the unique value of teaching and knowledge exchange that could only be transmitted through a personal interaction between students and teachers. Further research on online teaching, focusing on an adequate engagement of students and their satisfaction, is needed to understand how academics can better provide online course of medical statistics, biostatistics, and epidemiology allowing students to learn best in an online environment. In fact, the perceived good satisfaction of Italian academics of medical statistics in providing online teaching during the COVID‐19 outbreak does not necessarily imply an effective online teaching of statistics, but it is a step toward the implementation of online learning of medical statistics into the curriculum.

## CONFLICT OF INTERESTS

The authors have no conflicts of interest to declare.

## Supporting information

**Data S1.** Supporting information.

## Data Availability

Survey data that support the findings of this study are available from the corresponding author upon reasonable request.

## Appendix

**TABLE A1:** Descriptive statistics^*^ of selected items of the survey on online teaching and remote exams administered to the Italian academics of medical statistics in May 2020

Academic role and demographic data	
Number of respondents	94
Academic role	
Full professor	23 (24%)
Associate professor	39 (41%)
Assistant professor	27 (29%)
Adjunct professor	5 (5%)
Age (years)	
≤40	14 (15%)
41‐50	35 (37%)
51‐60	29 (31%)
≥61	16 (17%)
Gender	
Female	51 (54%)
Male	43 (46%)

Section on online teaching	
Previous experience^a^ with online teaching	
No	57 (61%)
Yes	37 (39%)
Current experience^a^ with online teaching	
No	6 (6%)
Yes	88 (94%)
Degree courses within which the academic delivered online teaching^b^, ^c^	
Single cycle degree in Medicine and Surgery	34 (39%)
Single cycle degree in Dentistry and Dental Prosthetics	15 (17%)
Bachelor's and master's degrees in Healthcare Professions	52 (59%)
First‐ and second‐level Master degrees, PhD Schools, and Schools of Medical Specialties	41 (47%)
Other courses	50 (57%)
Number of teaching hours^c^	
≤50	45 (51%)
>50	42 (48%)
Missing	1 (1%)
Median (IQR)	49 (30‐69)
Number of online teaching courses^c^	
≤2	51 (58%)
>2	36 (41%)
Missing	1 (1%)
Median (IQR)	2 (1‐7)
Number of students expected to attend the largest course the academic delivered online^c^	
≤100	54 (61%)
>100	32 (36%)
Missing	2 (2%)
Median (IQR)	100 (50‐120)
Online teaching modality^c^	
Asynchronous	21 (24%)
Synchronous	31 (35%)
Both	18 (20%)
Mixed	18 (20%)
Ever experienced technical difficulties with online teaching^c^	
No	69 (78%)
Yes	19 (22%)
Satisfaction with online teaching^c^	
<7/10	22 (25%)
≥7/10	66 (75%)
Median (IQR)	7 (7‐8)

Section on online exams	
Current experience^a^ with online exams	
No	3 (3%)
Not yet but I will	22 (23%)
Yes	69 (73%)
Students' number to be examined^d^	
≤100	55 (60%)
>100	36 (40%)
Median (IQR)	100 (60‐235)
Online exams modality^d^	
Oral only	20 (22%)
Online written only	26 (29%)
Oral and online written	37 (41%)
Other	8 (8%)
Need to create multiple rounds for the same exam^d^	
No	29 (32%)
Yes	49 (54%)
Do not know	7 (8%)
Missing	6 (7%)
Ever experienced technical difficulties with online exams^d^	
No	46 (51%)
Not yet delivered online exams	22 (24%)
Yes	19 (21%)
Missing	4 (4%)
Satisfaction with online exams^d^	
<7/10	25 (27%)
≥7/10	40 (44%)
Not yet delivered online exams	22 (24%)
Missing	4 (4%)
Median (IQR)	7 (6‐8)

*Percentages may not total 100 due to rounding.

^a^ Before the decree of the Presidency of the Council of Ministers of February 23, 2020, and subsequent which has ordered the suspension of in‐presence didactic activities.

^b^ The sum does not add up to the total since academics generally teach in more than one degree course.

^c^ Question to be compiled only by those who provided online teaching following the suspension of in‐presence didactic activities due to the decree of the Presidency of the Council of Ministers of February 23, 2020, and later.

^d^ Question to be compiled only by those who provided or will provide online exams following the suspension of in‐presence didactic activities due to the decree of the Presidency of the Council of Ministers of February 23, 2020, and later.

**TABLE A2:** Association between teaching variables and satisfaction with online teaching

	Fully satisfied^a^ with online teaching	RR (95% CI)	*P*
n	66/88 (75%)		
Age (years)			.60
≤40	8/12 (67%)	1 (Reference)	
41‐50	25/33 (76%)	1.14 (0.73‐1.77)	
51‐60	20/28 (71%)	1.07 (0.67‐1.70)	
≥61	13/15 (87%)	1.30 (0.83‐2.03)	
Gender			.54
M	32/41 (78%)	1 (Reference)	
F	34/47 (72%)	0.93 (0.73‐1.18)	
Academic role			.91
Assistant professor	19/25 (76%)	1 (Reference)	
Associate professor	28/36 (78%)	1.02 (0.80‐1.29)	
Full professor	15/23 (65%)	0.90 (0.67‐1.21)	
Adjunct professor	4/4 (100%)	‐	
Previous experience with online teaching			.53
No	41/53 (77%)	1 (Reference)	
Yes	25/35 (71%)	0.92 (0.72‐1.19)	
Teaching hours (number)			.57
≤50	33/45 (73%)	1 (Reference)	
>50	33/42 (79%)	1.07 (0.85‐1.36)	
Number of online teaching courses			.19
≤2	36/51 (71%)	1 (Reference)	
>2	29/36 (81%)	1.17 (0.93‐1.48)	
Number of students expected to attend the largest course you delivered online			.15
≤100	43/54 (80%)	1 (Reference)	
>100	21/32 (66%)	0.82 (0.62‐1.10)	
Online teaching modality			.18
Asynchronous	13/21 (62%)	1 (Reference)	
Synchronous	22/31 (71%)	1.15 (0.77‐1.72)	
Both	16/18 (89%)	1.44 (0.99‐2.09)	
Mixed	15/18 (83%)	1.35 (0.91‐2.00)	
Ever experienced technical difficulties with online teaching			.46
No	53/69 (77%)	1 (Reference)	
Yes	13/19 (68%)	0.89 (0.64‐1.24)	

Abbreviations: CI, confidence interval; RR, relative risk.

^a^ Satisfaction was defined as a score ≥ 7/10 at the question: “From 1 to 10, how much did you enjoy delivery online teaching?”

**TABLE A3:** Association between teaching variables and satisfaction with online exams

	Fully satisfied^a^ with online exams	RR (95% CI)	*P*
n	40/65 (62%)		
Age (years)			.49
≤40	6/11 (55%)	1 (Reference)	
41‐50	13/24 (54%)	0.99 (0.52‐1.91)	
51‐60	15/20 (75%)	1.38 (0.76‐2.50)	
≥61	6/10 (60%)	1.10 (0.53‐2.30)	
Gender			.07
M	22/30 (73%)	1 (Reference)	
F	18/35 (51%)	0.70 (0.48‐1.03)	
Academic role			.91
Assistant professor	13/20 (65%)	1 (Reference)	
Associate professor	17/27 (63%)	0.98 (0.70‐1.37‐)	
Full professor	8/16 (50%)	0.85 (0.56‐1.31)	
Adjunct professor	2/2 (100%)	‐	
Students' number to be examined			.56
≤100	22/37 (59%)	1 (Reference)	
>100	18/28 (64%)	1.12 (0.76‐1.66)	
Online exams modality			.36
Oral only	7/12 (58%)	1 (Reference)	
Online written only	15/21 (71%)	1.14 (0.75‐1.73)	
Oral and online written	15/29 (52%)	0.93 (0.60‐1.44)	
Need to create multiple rounds for the same exam			.87
No	15/23 (65%)	1 (Reference)	
Yes	24/40 (60%)	0.92 (0.62‐1.36)	
Do not know	1/2 (50%)	0.76 (0.19‐3.16)	
Ever experienced technical difficulties with online exams			<.01
No	36/46 (78%)	1 (Reference)	
Yes	4/19 (21%)	0.27 (0.11‐0.65)	

Abbreviations: CI, confidence interval; RR, relative risk.

^a^ Satisfaction was defined as a score ≥ 7/10 at the question: “From 1 to 10, how much did you enjoy delivery online exams?”

## References

[1] M. Bączek, M. Zagańczyk‐Bączek, M. Szpringer, A. Jaroszyński, and B. Wożakowska‐Kapłon, Students' perception of online learning during the COVID‐19 pandemic: A survey study of Polish medical students, Medicine (Baltimore) 100(7) (2021), e24821. 10.1097/MD.0000000000024821. 33607848 PMC7899848

[2] S. Baum and M. S. McPherson, The human factor: The promise & limits of online education, in Improving teaching: Strengthening the college learning experience, Vol 148, S. Baum and M. S. McPherson, Eds., American Academy of Arts and Sciences, Daedalus, Cambridge, 2019, 235–254. 10.1162/dead_e_01757.

[3] D. U. Bolliger and O. Wasilik, Factors influencing faculty satisfaction with online teaching and learning in higher education, Distance Educ 30(1) (2009), 103–116. 10.1080/01587910902845949.

[4] C. Dowling, J. M. Godfrey, and N. Gyles, Do hybrid flexible delivery teaching methods improve accounting students' learning outcomes? Acc Educ 12(4) (2003), 373–391. 10.1080/0963928032000154512.

[5] C. Gallo, F. Cavallo, and A. Bossi, Teaching medical statistics to undergraduate medical students: What is taught and what is really useful for a medical professional? A report of the Education Committee of the Italian Society of Medical Statistics and Clinical Epidemiology (SISMEC), Epidemiol Biostat Public Health 16(3) (2019), 1–10. 10.2427/13205.

[6] C. S. González‐González, A. Infante‐Moro, and J. C. Infante‐Moro, Implementation of E‐proctoring in online teaching: A study about motivational factors, Sustainability 12(8) (2020), 3488. 10.3390/su12083488.

[7] K. Hamann, R. A. Glazier, B. M. Wilson, and P. H. Pollock, Online teaching, student success, and retention in political science courses, Eur Political Sci (2020). 10.1057/s41304-020-00282-x.

[8] D. Harrington, Teaching statistics: A comparison of traditional classroom and programmed instruction/distance learning approaches, J Soc Work Educ 35(3) (1999), 343–352. 10.1080/10437797.1999.10778973.

[9] Italian Ministry of Education, University and Research . Official database of Italian Academics, April 30, 2020, avilable at https://cercauniversita.cineca.it/php5/docenti/cerca.php

[10] C. H. McLaren, A comparison of student persistence and performance in online and classroom business statistics experiences, Decis Sci J Innov 2(1) (2004), 1–10. 10.1111/j.0011-7315.2004.00015.x.

[11] J. P. Merisotis and R. A. Phipps, What's the difference?: Outcomes of distance vs traditional classroom‐based learning, Change 31(3) (1999), 12–17. 10.1080/00091389909602685.

[12] R. W. Morris, Does EBM offer the best opportunity yet for teaching medical statistics? Stat Med 21(7) (2002), 969–977. 10.1002/sim.1126. 11921003

[13] A. Y. Ni, Comparing the effectiveness of classroom and online learning: Teaching research methods, J Public Aff Educ 19(2) (2013), 199–215. 10.1080/15236803.2013.12001730.

[14] Società Italiana di Statistica Medica ed Epidemiologia Clinica (SISMEC) . Official website of the SISMEC, May 25, 2020, available at https://www.sismec.info

[15] D. Xu and S. S. Jaggars, Performance gaps between online and face‐to‐face courses: Differences across types of students and academic subject areas, J High Educ 85(5) (2014), 633–659. 10.1080/00221546.2014.11777343.

[16] D. Yang, Instructional strategies and course design for teaching statistics online: Perspectives from online students, Int J Stem Educ 4 (2007), 34. 10.1186/s40594-017-0096-x. PMC6310393 30631690

