# Supplementary material for: Satisfaction with online teaching of medical statistics during the COVID‐19 pandemic: A survey by the Education Committee of the Italian Society of Medical Statistics and Clinical Epidemiology
Source: Teach Stat. 2021 Jul 22;43(3):129–39. doi: 10.1111/test.12286 (PMC8447082; doi:10.1111/test.12286)
Supplement: Supplementary file 1 — Data S1. Supporting information. [file TEST-43-129-s001.docx]

**Supplementary Table 1.** Survey on online teaching and online exams administered to the Italian academics of medical statistics in May 2020.

| Role and demographic data | | |
| --- | --- | --- |
| Academic role | - Full professor - Associate professor - Assistant professor - Adjunct professor - Other [please specify] | |
| Age (years) | - ≤40 - 41-50 - 51-60 - ≥61 | |
| Gender | - Female - Male | |
| Section on online teaching | | |
| In the past^†^, did your university admit the possibility of providing online teaching? | | - Yes - No |
| In the past^†^, have you ever delivered online teaching? | | - Yes - No |
| Are you providing online teaching following the suspension of in-presence didactic activities due to the decree of the Presidency of the Council of Ministers of 23 February 2020 and later? | | - Yes - No |
| How much had you dedicate yourself to study in deep modalities to deliver online teaching? | | - Nothing at all - A little - A moderate amount - A lot |
| Has your university made available a technical and IT assistance service support for teachers involved in the provision of online teaching? | | - Yes - No |
| Have you made use of this technical and IT assistance service? | | - Yes - No |
| Which degree course do the teachings you are delivering online belong to? (multiple answers are possible) | | - Single cycle degree in Medicine and Surgery - Single cycle degree in Dentistry and Dental Prosthetics - Single cycle degree in Veterinary Medicine - Single cycle degree in Pharmacy - Bachelor's and master's degrees in Healthcare Professions - Bachelor's and Master's degrees in Motor Sciences - Bachelor's degrees in Biotechnology and Biological Sciences - Master's Degrees in Biology and Medical Biotechnology, Veterinary and Pharmaceuticals Sciences - Bachelor's degrees in Statistics - Master's degrees in Statistical, Actuarial and Financial Sciences - 1^st^ and 2^nd^ level Master degrees, PhD Schools and Schools of Medical Specialties |
| How many teachings are you delivering online? | | [Open field] |
| Overall, how many hours of online teaching are you providing? | | [Open field] |
| How many students are expected to attend the largest teaching you are delivering online? | | [Open field] |
| Is online teaching leading to a reduction of the didactic program? | | - Yes - No - Don’t know |
| How did you share the teaching material (slides, articles, code, etc.) with the students? | | - By uploading it on the University's LMS (“Learning Management System”) platform - Through clouding services (DropBox, OneDrive, Google Drive, etc ..) - By e-mail |
| Which modality did you choose to deliver online teaching? | | - Asynchronous (only video lessons uploaded on the University LMS platform) - Synchronous (only live lessons in virtual classrooms called by videoconference) - Both (live lessons in virtual classrooms recorded and then uploaded to the University LMS platform) - Mixed (some live lessons and some video lessons uploaded on the University LMS platform) |
| If you chose to use the asynchronous modality, which LMS (“Learning Management System”) platform/services are you using to deliver online teaching? (multiple answers are possible) | | - Moodle (through Kaltura media-server or other plug-in software) - Blackboard Learn - Edmodo - Ilias - Google Suite for Education - Office 365 Educational Suite - Other [please specify] |
| If you chose to use the synchronous modality, which software are you using to deliver online teaching? (multiple answers are possible) | | - Google Meet / Hanghouts - Microsoft Teams - Blackboard Collaborate - Skype - Zoom - WebEx - GoToMeeting - Jitsi - BigBlueButton - Adobe Connect - SkyMeeting - Other [please specify] |
| Have you dealt with, or are you planning to deal with statistical/epidemiological topics related to the Covid-19 epidemic within the online teachings you are providing? | | - Yes - No - Don’t know |
| How was your experience with online compared to traditional teaching? | | - Less effective - Comparable - More effective - Don’t know |
| Have you experienced technical difficulties with online teaching? | | - Yes - No |
| If so, what kind of technical problems have you experienced? (multiple answers are possible) | | - Connection problems (e.g. overload on the network and/or on the University LMS platform, interruptions on the connection, etc.) - Students’ difficulties in configuring the connection to the online lessons - Lack of knowledge and/or students’ difficulties with software platforms used to deliver online teaching - Other [please specify] |
| From 1 to 10, how much did you enjoy delivering online teaching? | | [1 2 3 4 5 6 7 8 9 10 ] |
| Use the space below if you want to share additional consideration on online teaching | | [Open field] |
| Section on online exams | | |
| Are you administering online exams following the suspension of in-presence didactic activities due to the decree of the Presidency of the Council of Ministers of 23 February 2020 and later? | | - Yes - No - Not yet but I will |
| Has your university issued a regulation covering technical aspects (modalities of connection with the examinees, identity verification, etc.) on the modalities to delivery online exams? | | - Yes - No - Don’t know |
| Overall, how many students did you examine, are you examining, or do you expect to examine online? | | [Open field] |
| Which modality are you using or are you planning to use to deliver online exams? (multiple answers are possible) | | - Oral - Traditional written exam with online control of the examinees - online administered quiz with online control of the examinees - Homeworks prepared by the examinees - I have not yet decided - Other [please specify] |
| If you chose to deliver written exams or online quizzes, have you used or are you planning to use an “e-proctoring” software (e.g. Respondus, Edx etc.) officially supplied by the university? | | - Yes - No - I would have used it if available - I don't know what an "e-proctoring" software is |
| If you chose to deliver written exams or online quizzes, have you used or will you use a lock-down browser (e.g. Safe Exam Browser) integrated into the university LMS (“Learning Management System”) platform? | | - Yes - No - I don't know what a lock-down browser is |
| Have you found or do you expect to find yourself forced to create multiple rounds for the same exam due to the high number of examinees? | | - Yes - No - I don’t know, I have not yet delivered online exams |
| Which software are you using to connect with the examinees to deliver online exams? (multiple answers are possible) | | - Google Meet / Hanghouts - Microsoft Teams - Blackboard Collaborate - Skype - Zoom - WebEx - GoToMeeting - Jitsi - BigBlueButton - Adobe Connect - SkyMeeting - Other [please specify] |
| Which modality are you using to connect with the examinees to deliver online exams? (multiple answers are possible) | | - Examinees connected only through a PC equipped with a webcam - Examinees connected only through a smartphone that frames the examinee’s workstation - Examinees connected only through a smartphone - Examinees connected through a PC equipped with a webcam and also with a smartphone - I have not yet decided - Other [please specify] |
| Have you verified or are you planning to verify online the identity of the examinees according to the university’s procedures? | | - Yes - No - Don’t know, I have not yet delivered online exams |
| Have you ever had to reprimand a student because he/she didn't follow the university’s rules of conduct for taking online exams? | | - Yes - No - I have not yet delivered online exams |
| How difficult was your experience with online exams? | | - Not at all - A little - A moderate amount - A lot - I have not delivered online exams so far |
| How were student’s evaluations with online compared to traditional exams? | | - Lower - Comparable - Higher - Don’t know - I have not yet delivered online exams |
| Have you experienced any difficulties with online exams? | | - Yes - No - I have not yet delivered online exams |
| If so, what kind of problems have you experienced? (multiple answers possible) | | - Difficulty and/or doubts in verifying the identity of the examinees - Connection problems (e.g. overload on the network and/or on the University LMS platform, interruptions on the connection, etc.) - Students’ difficulties in configuring the connection to the online exams - My lack of knowledge of the functionalities of the software platform to deliver online exams (including "e-proctoring" software) - Difficulty of examinees in using the software platform used to deliver the online exam - Lack of adequate software and hardware equipment of the examinees - Other [please specify] |
| From 1 to 10, how much did you enjoy delivering online exams? | | [1 2 3 4 5 6 7 8 9 10 ] |
| Use the space below if you want to share additional consideration on online exams | | [Open field] |
| Section on perspectives of online teaching | | |
| Would you provide online teaching in the future? | | - Yes - No - Don’t know |
| Would you change the modality you used to deliver online teaching in the future? | | - Yes - No - Don’t know |
| Would you change the modality you used to deliver online exams in the future? | | - Yes - No - Don’t know |
| From 1 to 10, how effective do you think online teaching of statistics is? | | [1 2 3 4 5 6 7 8 9 10 ] |

^†^ before the decree of the Presidency of the Council of Ministers of 23 February 2020 and subsequent which has ordered the suspension of in-presence didactic activities.
